# Post-mortem gene expression of calcium channels Cav1.2 and Cav1.3 in schizophrenia

**DOI:** 10.1007/s00406-022-01482-w

**Published:** 2022-09-06

**Authors:** Andrea Schmitt, Stefanie Uhrig, Rainer Spanagel, Martina von Wilmsdorff, Janos L. Kalman, Thomas Schneider-Axmann, Peter Falkai, Anita C. Hansson

**Affiliations:** 1 Department of Psychiatry and Psychotherapy, University Hospital, LMU Munich, Munich, Germany; 2 Laboratory of Neuroscience (LIM27), Institute of Psychiatry, University of Sao Paulo, São Paulo SP, Brazil; 3 Medical Faculty Mannheim, Institute for Psychopharmacology at Central Institute for Mental Health, University of Heidelberg, Heidelberg, Germany; 4 Department of Psychiatry and Psychotherapy, Medical Faculty, Heinrich-Heine-University, Düsseldorf, Germany; 5 Institute of Psychiatric Phenomics and Genomics (IPPG), University Hospital, LMU Munich, Munich, Germany; 6 Max Planck Institute of Psychiatry, Munich, Germany

Schizophrenia is a severe neuropsychiatric disorder with a heritability of 60–80%, and is associated with an unfavorable outcome including cognitive impairment, in more than half of the patients. Large-scale genome-wide association studies (GWAS) have identified common variant associations at 287 distinct genomic loci, which are concentrated in genes related to neuronal development and function with prominent enrichments at the synapse [1]. Calcium voltage-gated channel subunit alpha1 C (CACNA1C) and calcium voltage-gated channel subunit alpha1 D (CACNA1D) genes, which encode the L-type calcium channel isoforms Cav1.2 and Cav1.3 respectively, are important regulators of calcium influx into cells and critical for normal brain development and plasticity. They have been described to be involved in the modulation of the accumbal dopamine signaling pathway, synaptic transmission of auditory stimuli and synaptic plasticity of neutral and aversive learning and memory processes [2]. GWAS studies have described an association of SNPs within the CACNA1C gene (but not CACNA1D) and schizophrenia susceptibility [1]. Furthermore, risk-associated genetic variation in CACNA1C in healthy human participants was associated with impairments in reversal learning and decreased expression of prefrontal brain-derived neurotrophic factor (BDNF) [3]. The neurobiological consequences of genetic variation in CACNA1C and CACNA1D genes should, however, be elucidated in more detail. Therefore, in this study, we investigated the mRNA expression of the two isoforms Cav1.2 and Cav1.3 in post-mortem prefrontal, temporal, cerebellar and caudate brain regions in schizophrenia patients compared to healthy controls.

Post-mortem brain samples were obtained as described in Uhrig et al. [4], and the use was approved by the Ethics Committee of the Faculty of Medicine, University of Heidelberg, Germany (009–238-MA). Briefly, brains from nine in-patients with DSM-IV residual schizophrenia (5 males, 4 females; mean (standard deviation, SD) age 68.22 (14.99) years; post-mortem interval (PMI) 20.56 (10.71) hours; duration of disease 41.44 (12.38) years; last dose of antipsychotic treatment in chlorpromazine equivalents 283.33 (252,45) mg; cumulative dose of antipsychotic treatment in chlorpromazine equivalents during the last ten years 3.44 (2.85) kg) were obtained from the Department of Neuropathology, Psychiatric Center Nordbaden, Wiesloch, Germany. Post-mortem brain samples from six healthy controls (5 males, 1 female; mean (SD) age 61.83 (16, 76) years; PMI 15.76 (5, 61) hours) were obtained from autopsies performed at the Institute of Neuropathology, University of Heidelberg, Germany. Gray matter blocks of the left anterior prefrontal cortex (Brodmann area 10, BA10), left posterior medial temporal cortex (Brodmann area 21, BA21), left nucleus caudatus (N. caudatus) and the right cerebellar posterior superior vermis (vermis) were dissected, snap-frozen in liquid nitrogen-cooled isopentane, and stored at − 80 °C until use. As described previously [4], two groups of ten male Sprague Dawley rats were fed with pellets mixed with antipsychotics. From postnatal day (PD) 85 until the end of a 12-week treatment period, the first group was fed with haloperidol at a dose of 1 mg/kg bodyweight (BW)/day and the second group with clozapine at a dose of 20 mg/BW/day. The third served as a control group and received no antipsychotic treatment. The animals were anaesthetized by pentobarbital, sacrificed and brains were removed, frozen and stored at − 80 °C. Tissues of brain regions (anterior cingulate cortex, pre-limbic cortex, caudate putamen, hippocampal sub-regions cornu ammonis (CA) 1 and 3, and dentate gyrus) were obtained by micro-dissection. All animal experiments were approved by the local animal care committee (AZ 9.93.2.10.34.07.227). mRNA was extracted and analyzed as previously described by quantitative Real-Time PCR (qRT-PCR) [4]. In each sample, the quality of the RNA was assessed by bio-analyzer and only mRNA with RNA integrity number (RIN) above 6 was included in the analyses. Triplicates of samples were assayed with quantitative real-time polymerase chain reaction (qRT-PCR, primer sequences for CACNA1C: Forward primer 5’-GCAGGAGTACAAGAACTGTGAGC-3’, reverse primer 5’-CGAAGTAGGTGGAGTTGACCAC-3’, gene reference sequence: NM_199460; primer sequences for CACNA1D: Forward 5’-CTTCGACAACGTCCTCTCTGCT-3’, reverse 5’-GCCGATGTTCTCTCCATTCGAG-3’, gene reference sequence: NM_000720.3; primer sequences for *GAPDH*: Forward 5’- ATGAGAAGTATGACAACAGCCT-3’, reverse 5’- AGTCCTTCCACGATACCAAAGT-3 ‘, gene reference sequence: NM_002046.4).

Relative quantification was performed according to the deltaCt (dCt) method [4], whereby the human housekeeping gene glyceraldehyde-3-phosphate dehydrogenase (GAPDH) was used as the internal normalizer (dCt = Ct_Cav_–Ct_Gapdh_). ddCt values indicated changes in schizophrenia patients (ddCt = dCt_Controls_–dCt_Patients_) versus controls. The significance level was set at *α* = 0.05, and all tests were two-tailed. Statistical analyses were performed with IBM SPSS statistics 22. All data are presented as mean ± SEM. Results from the Kolmogorov–Smirnov test suggested a normal distribution of the data, allowing analysis by parametric tests. The human post-mortem samples were analyzed by a region-wise ANCOVA of the dCt values adjusted for age and PMI. In the total sample and separately for schizophrenia patients and controls, Pearson’s correlations were used to test for associations between both Cav1.2 and Cav1.3 gene expression and age at death, PMI, dose of antipsychotic treatment in CPE and disease duration. In the rat samples, a region-wise one-way ANOVA, if applicable followed by Fisher’s LSD test, was performed. Data are expressed as mean ± SEM. Since this was an explorative study with small group sizes, results are presented without error probability correction. If a Bonferroni adjustment of the type I error probability had been applied, no significant differences would have remained between schizophrenia patients and controls. However, if error probability had been adjusted, the power for detecting existing mean differences would have been too low.

**Fig. 1:**
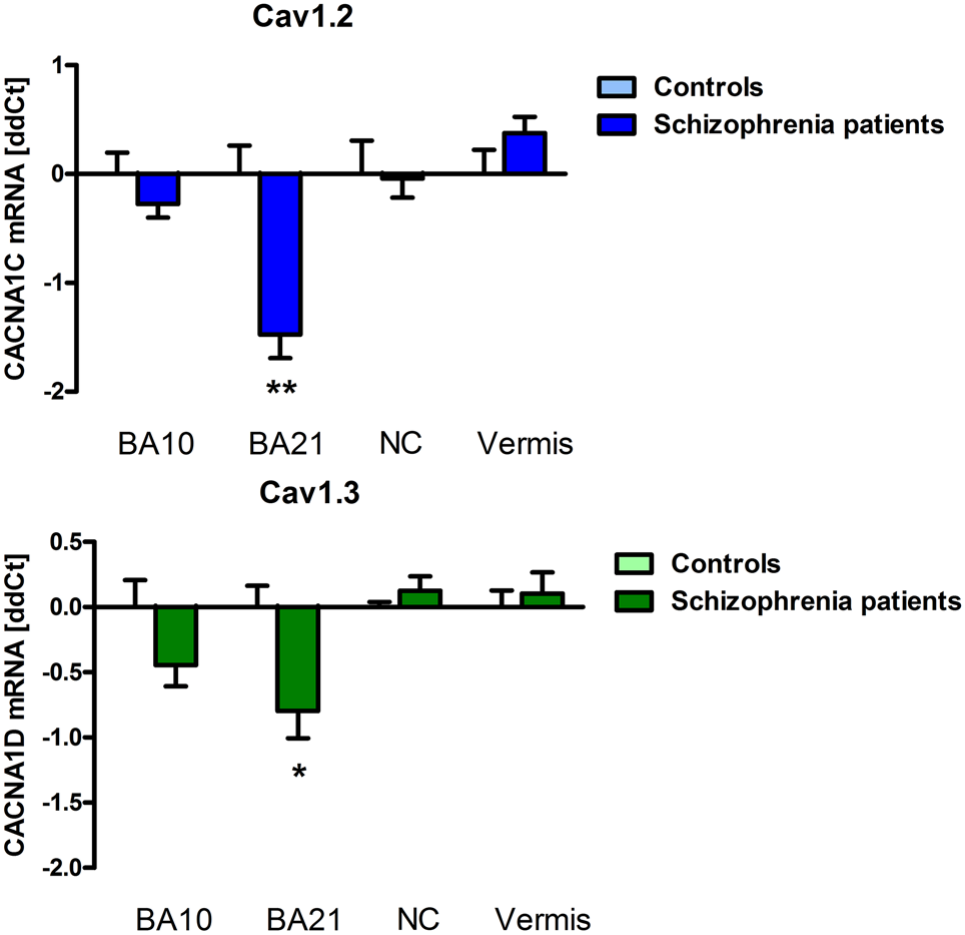
qRT-PCR revealed downregulated expression of CACNA1C (Cav1.2; blue bars) and CACNA1D (Cav1.3; green bars) in the temporal cortex (BA21) of schizophrenia patients compared to control subjects. Bars show ddCt values, with GAPDH as internal normalizer, as mean ± SEM. Brodmann Area *BA*, nucleus caudatus *NC*, cerebellar vermis, vermis

We found a significant downregulation of Cav1.2 mRNA in schizophrenia patients (8.16 ± 0.21 dCt) compared to controls (6.68 + / − 0.26 dCt) in the left BA21 (F(1, 7) = 14.78), *P* = 0.0063). Additionally, we detected a significant reduction of Cav1.3 in schizophrenia patients (5.15 + / − 0. 21 dCt) in left BA21 (controls 4.35 + / − 0.16 dCt; F(1, 9) = 5.84, *P* = 0.046) (Fig. 1). GAPDH Ct values did not differ between the groups. No significant differences between patients and controls were found in BA10, nucleus caudatus and cerebellar vermis, or in the rats treated with haloperidol or clozapine compared the untreated controls in any of the analyzed regions. In schizophrenia patients, Cav1.2 expression in BA21 (*r* = 0.871, *P* = 0.024) and N. caudatus (*r* =  − 0.895, *P* = 0.006) correlated with age. In healthy controls, Cav1.3 expression correlated with age in BA21 (*r* =  − 0.889, *P* = 0.044). Duration of disease correlated with Cav1.2 expression in BA21 (r = 0.933, P = 0.007) and N. caudatus (*r* =  − 0.769, *P* = 0.043). PMI and dose of antipsychotic medication in CPE did not correlate with mRNA in either group or the entire sample. Gender had no influence on the results.

In summary, this post-mortem study shows a downregulation of Cav1.2 mRNA and reduction of Cav1.3 in the left posterior medial temporal cortex in schizophrenia patients. Based on results from our animal model and the correlation analyses, these findings are unlikely affected by antipsychotic treatment. In a combined neuroimaging, genetic association and gene expression study in healthy volunteers, the risk-associated single-nucleotide polymorphism (SNP) rs1006737 in CACNA1C predicted increased prefrontal activity during executive cognition and increased mRNA expression in post-mortem prenatal human prefrontal cortex [5]. However, these samples were collected during neurodevelopment, and no post-mortem brains from schizophrenia patients have been investigated so far. In contrast to our results in adult patients, in induced human neurons from healthy volunteers with the CACNA1C homozygous risk genotype SNP rs1006737, an increased mRNA expression of Cav1.2 has been demonstrated compared to the non-risk genotype [6]. However, in patients with bipolar disorder, the SNP rs1006737 risk allele (A) was associated with reduced expression of CACNA1C. The association was present in multiple exons as well as transcripts of this gene in cerebellum but not in parietal cortex. This finding is also present for rs1024582, the CACNA1C SNP recently associated with multiple psychiatric disorders and its risk allele (A) is also associated with significantly decreased cerebellar expression of the same CACNA1C probes [7]. In transgenic animal models, developmental lesion of CACNA1C induces cognitive deficits, hyperactivity and anxiety in contrast to heterozygosity or post-embryonic knockout models, which leads to reduced anxiety and locomotion (for review see 8). Additionally, heterozygous global deletion of CACNA1C during development has been shown to increase susceptibility to chronic social defeat stress [8], which also plays a major cross-species role, just as in childhood trauma in schizophrenia patients. Rats with heterozygous lesion of CACNA1C show deficits in reversal learning. In healthy humans, risk-associated genetic variation in CACNA1C was also associated with impairments in reversal learning [3]. A recent meta-analysis demonstrated an effect of CACNA1C rs1006737 polymorphism with cognitive function in patients with schizophrenia and bipolar disorder [9]. This issue presents an article by Guardiola-Ripoll et al. [10] who detected a significant CACNA1C x ZNAF804A interaction on working memory-based functional response in the ventral caudate, the left superior and inferior orbitofrontal gyrus, the left superior temporal pole and the ventral–anterior insula. Future studies should investigate in more detail the neurobiological background of CACNA1C and CACNA1D risk polymorphisms in schizophrenia with a special focus on synaptic plasticity and cognitive impairment. This may pave the way for developing new treatment targets for schizophrenia.
